# Preparation and Evaluation of Enteric-Coated Chitosan Derivative-Based Microparticles Loaded with Salmon Calcitonin as an Oral Delivery System

**DOI:** 10.3390/ijms17091546

**Published:** 2016-09-13

**Authors:** Hiraku Onishi, Ayako Tokuyasu

**Affiliations:** Department of Drug Delivery Research, Hoshi University, 2-4-41 Ebara, Shinagawa-ku, Tokyo 142-8501, Japan; ri300080-1347@tbz.t-com.ne.jp

**Keywords:** chitosan-4-thio-butylamidine conjugate microparticles, Eudragit L100-coated microparticles, salmon calcitonin, particle feature, in vitro release, in vivo efficacy, oral drug delivery system

## Abstract

Background: The production of protein drugs has recently increased due to advances in biotechnology, but their clinical use is generally limited to parenteral administration due to low absorption in non-parenteral administration. Therefore, non-parenteral delivery systems allowing sufficient absorption draw much attention. Methods: Microparticles (MP) were prepared using chitosan-4-thio-butylamidine conjugate (Ch-TBA), trimethyl-chitosan (TMC), and chitosan (Ch). Using salmon calcitonin (sCT) as a model protein drug, Ch-TBA-, Ch-TBA/TMC (4/1)-, and Ch-based MP were produced, and their Eudragit L100 (Eud)-coated MP, named Ch-TBA-MP/Eud, Ch-TBA/TMC-MP/Eud, and Ch-MP/Eud, respectively, were prepared as oral delivery systems. These enteric-coated microparticles were examined in vitro and in vivo. Results: All microparticles before and after enteric coating had a submicron size (600–800 nm) and micrometer size (1300–1500 nm), respectively. In vitro release patterns were similar among all microparticles; release occurred gradually, and the release rate was slower at pH 1.2 than at pH 6.8. In oral ingestion, Ch-TBA-MP/Eud suppressed plasma Ca levels most effectively among the microparticles tested. The relative effectiveness of Ch-TBA-MP/Eud to the intramuscular injection was 8.6%, while the sCT solution showed no effectiveness. Conclusion: The results suggest that Eud-coated Ch-TBA-based microparticles should have potential as an oral delivery system of protein drugs.

## 1. Introduction

Bioactive proteins and peptides, such as insulin or interferon, have recently been produced in large amounts due to advances in biotechnology [1,2]. Antibody drugs are highly effective in the treatment of chronic inflammatory diseases [3]. Following their oral administration, peptide drugs are generally degraded rapidly by hydrolytic enzymes and have weak permeability through the intestinal membrane; therefore, they are commonly administered via a parenteral route [4]. Although the parenteral administration of peptide drugs achieves pharmacological responses, repeated injections are stressful for patients due to pain, and needle-stick injuries may lead to infection.

**Figure 1:**
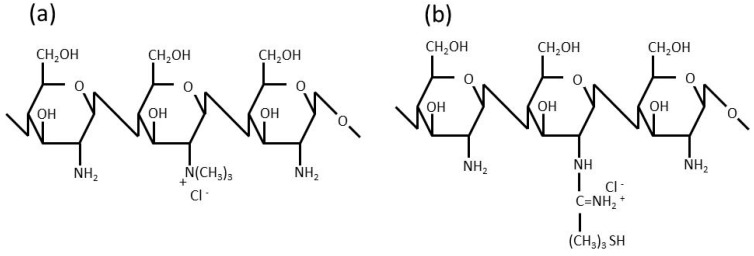
Chemical structures of (**a**) trimethyl-chitosan (TMC); and (**b**) chitosan-4-thio-butylamidine conjugate (Ch-TBA).

In order to overcome the drawbacks of the parenteral administration of peptide drugs, their absorption via various mucosal membranes has been investigated [5,6,7]. Peptide drugs are reportedly absorbed into the systemic circulation to a certain extent through the oral mucosa, rectal mucosa, and nasal mucosa. In mucosal absorption studies, various techniques to enhance absorption have been examined, and the underlying mechanisms have been studied from physicochemical and physiological viewpoints [8,9,10,11,12]. One of these absorption enhancement methods involves the use of permeation-enhancing polymers [13,14,15,16]. Chitosan (Ch) and its derivatives have recently been attracting attention because they promote permeation and induce less damage to mucosal membranes [17,18,19,20,21]. Trimethyl-chitosan (TMC) [22,23,24,25,26] and chitosan-4-thio-butylamidine conjugate (Ch-TBA) [27,28,29,30,31], shown in Figure 1, strongly enhance permeation. Ch only promotes permeation under acidic conditions because it is cationized under these conditions. On the other hand, TMC enhances permeation under acidic, neutral, and basic conditions because of the cationizing characteristics of its quaternary amine in a wide range of pH. TMC was previously reported to exhibit equivalent or higher permeation-enhancing potential for some peptide drugs, in which the opening of the tight junction of the mucosal membrane was found to be involved in the enhancements observed in permeation [26]. Ch-TBA is considered to contribute to tight junction opening by increasing the amount of reduced glutathione (GSH), which inhibits protein tyrosine phosphate activity [11,28].

In the present study, salmon calcitonin (sCT) was selected as the model peptide drug. Ch, Ch-TBA, and Ch-TBA/TMC microparticles (MP) containing sCT, named Ch-MP, Ch-TBA-MP, and Ch-TBA/TMC-MP, respectively, were produced. Ch-MP, Ch-TBA-MP, and Ch-TBA/TMC-MP were coated with the enteric polymer, Eudragit L100. The in vitro characteristics and in vivo effectiveness of the resultant enteric-coated microparticles, called Ch-MP/Eud, Ch-TBA-MP/Eud, and Ch-TBA/TMC-MP/Eud, respectively, were investigated and evaluated. 

## 2. Results and Discussion

### 2.1. Synthesis and Characteristics of TMC and Ch-TBA

The ratio of the *N*-trimethyl group in TMC was examined based on the proton nuclear magnetic resonance (NMR) spectra which were obtained using D_2_O and 1% (*v/v*) CD_3_COOD in D_2_O as solvents. As the details of the signal assignment had been reported before [32], the proton signal found at a 0.9 ppm low magnetic field against the HDO proton signal was assigned as the protons of N^+^(CH_3_)_3_, which was based on the report by Sieval et al. [22]. The substitution degree was calculated by comparing the integrated intensity of the *N*-trimethyl proton signal (3.7–3.8 ppm) with that of the C1 proton signal (4.8–6 ppm). The degree of *N*-trimethylation was 56% (mol/mol) of the glucosamine unit.

The substitution degree of 4-thio-butyl-amidine to Ch was investigated in Ch-TBA by measuring the content of the thiol (-SH) group, which was evaluated using the 2,2′-dithiodipyridine (2-PDS) method [33]. The measurement of thiol groups with 5,5′-dithio-bis(2-nitrobenzoic acid) (DTNB), named the DTNB method, has been shown to provide inexact or irreproducible results, whereas the 2-PDS method achieves good and reproducible results because it is applicable to a wide pH range from weakly acidic to neutral conditions [33]. The content of the thiol group was 798.8 µmol/g polymer, and Ch-TBA dissolved well at pH 4.5.

### 2.2. Formulations and Particle Characteristics of Ch-TBA-MP, Ch-TBA/TMC-MP, and Ch-MP

The formulations and particle characteristics of the microparticles before the Eudragit coating are shown in Table 1. Ch-MP formed well with the W/O emulsification-evaporation method using sorbitan sesquioleate (SO-15)-containing liquid paraffin, which has been reported to produce chitosan microparticles containing other drugs [34,35,36]. Ch-TBA-MP also formed well with this method. Ch-TBA/TMC-MP were obtained at a Ch-TBA/TMC ratio of 4/1, but could not be produced well at 1/1 or 1/4. 

The solubility characteristics of Ch-TBA in aqueous media were similar to those of Ch; they were only soluble in acidic media. Therefore, Ch-TBA-MP were considered to form as well as Ch-MP. On the other hand, TMC is highly water-soluble because it is cationized at a wide pH range from acidic to basic conditions. Therefore, repulsion may have occurred between TMC molecules during the formation process of microparticles with TMC. Furthermore, since TMC is highly water-soluble, microparticles containing TMC may be subjected to agglutination without complete dryness. This may explain why Ch-TBA/TMC-MP did not form well when TMC was used. Well-formed Ch-TBA/TMC(4/1)-MP were used as Ch-TBA/TMC-MP in subsequent experiments.

**Figure 2:**
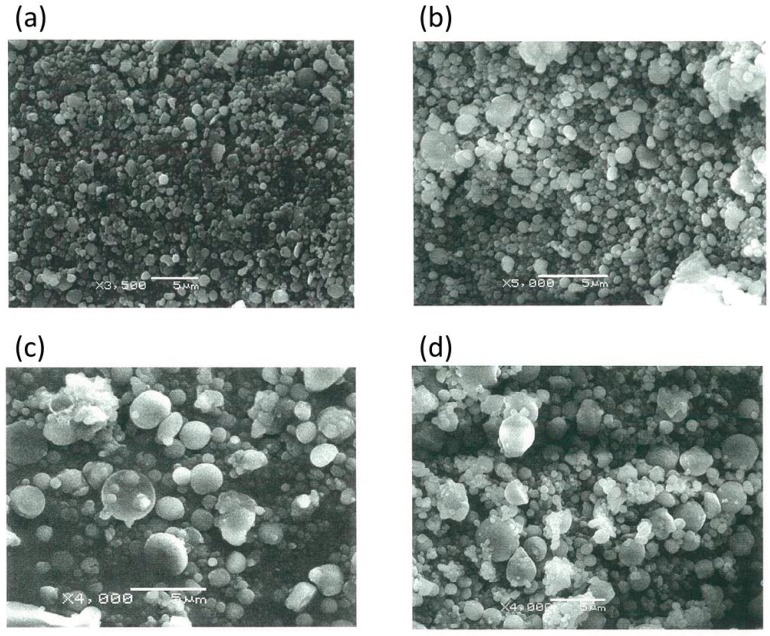
Scanning electron micrographs of (**a**) Ch-TBA-MP; (**b**) Ch-TBA/TMC-MP *; (**c**) Ch-TBA-MP/Eud; and (**d**) Ch-TBA/TMC-MP/Eud *. * Ch-TBA/TMC(4/1)-MP were used. The length of the white bar = 5 µm. MP: microparticles.

Scanning electron microscopy (SEM) images of well-formed Ch-TBA-MP and Ch-TBA/TMC-MP in an almost spherical shape are shown in Figure 2a,b. Ch-TBA had a mean size of 698 nm with a size distribution between 200 and 1500 nm. Ch-TBA/TMC-MP had a mean size of 778 nm with a size distribution between 200 and 1500 nm. Both types of microparticles mainly had a submicron size (<1000 nm). Ch-MP exhibited similar particle features for shape and size (mean size = 654 nm) (SEM image, not shown).

### 2.3. Particle Characteristics and sCT Contents of Ch-TBA-MP/Eud, Ch-TBA/TMC-MP/Eud, and Ch-MP/Eud 

Ch-TBA-MP, Ch-TBA/TMC-MP, and Ch-MP were coated with Eudragit L100 using a similar emulsification-evaporation method. Ch-MP formed well into Eudragit L100-coated Ch-MP. SEM images of Ch-TBA-MP/Eud and Ch-TBA/TMC-MP/Eud are shown in Figure 2c,d. They had an almost spherical shape. Their particle sizes were markedly larger than those of Ch-TBA-MP and Ch-TBA/TMC-MP. The mean sizes of Ch-TBA-MP/Eud and Ch-TBA/TMC-MP/Eud were 1510 and 1400 nm, respectively (Table 2). Sizes were distributed between 300 and 5000 nm. These results indicated that Ch-TBA-MP and Ch-TBA/TMC-MP were coated well with Eudragit L100. Ch-MP, produced in a similar manner, had a spherical shape and mean size of 1350 nm (SEM image, not shown).

The contents of sCT were investigated in Ch-TBA-MP/Eud, Ch-TBA/TMC-MP/Eud, and Ch-MP/Eud. The results obtained are shown in Table 2. Ch-MP/Eud showed the highest content at 5.4 ± 0.8 µg/mg, followed by Ch-TBA-MP/Eud at 4.1 ± 1.2 µg/mg and Ch-TBA/TMC-MP/Eud at 2.7 ± 0.2 µg/mg. As stated above, the addition of TMC tended to disturb the formation of Ch-TBA/TMC-MP, the incorporation of sCT may have decreased. As sCT is a cationic protein, the use of TMC with a high N^+^(CH_3_)_3_ substitution degree (56% mol/mol) may have suppressed the incorporation of the sCT.

### 2.4. In Vitro Release of sCT from Ch-TBA-MP/Eud, Ch-TBA/TMC-MP/Eud, and Ch-MP/Eud

**Figure 3:**
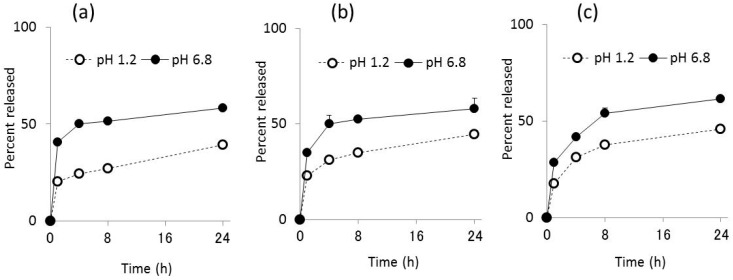
Release profiles of (**a**) salmon calcitonin (sCT) from Ch-TBA-MP/Eud; (**b**) Ch-TBA/TMC-MP/Eud *; and (**c**) Ch-TBA-MP/Eud. Each point represents the mean ± standard deviation (S.D.) (*n* = 3). * Ch-TBA/TMC(4/1)-MP were used.

The release of sCT from Ch-TBA-MP/Eud, Ch-TBA/TMC-MP/Eud, and Ch-MP/Eud was investigated under gastric pH conditions (JP 16 1st medium, pH 1.2) and intestinal pH conditions (JP 2nd fluid, pH 6.8). The release profiles obtained are shown in Figure 3. Release patterns were similar among all microparticles, whereas release rates differed to some extent. The initial release of sCT from Ch-TBA-MP was greater than that from the other microparticles. The release rate in the later phase was larger in the order of Ch-MP/Eud > Ch-TBA/TMC-MP/Eud > Ch-TBA-MP/Eud at pH 1.2 and 6.8. 

In the JP16 first fluid (pH 1.2), the initial burst of sCT was similar among the microparticles tested, with a release percentage of approximately 20%. sCT was then released gradually, with mean release percentages reaching 39%, 45%, and 46% at 24 h for Ch-TBA-MP/Eud, Ch-TBA/TMC-MP/Eud, and Ch-MP/Eud, respectively. 

Under intestinal conditions at pH 6.8, the amount of sCT released was greater than that at pH 1.2. Ch-TBA-MP/Eud, Ch-TBA/TMC-MP/Eud, and Ch-MP/Eud had mean release percentages of 40%, 35%, and 28%, respectively. After being incubated for 24 h, approximately 60% of sCT was released from all microparticle types. 

Thus, the Eud coating protected the inside microparticles and suppressed the release of sCT at the gastric pH. Although Ch-TBA and Ch are subjected to swelling or dissolution at pH 1.2, the release was suppressed for a long time, indicating the Eud coating functioned well in the protection of the inside microparticles. Furthermore, since the Eud layer dissolved at pH 6.8, sCT was released more rapidly than in the case of pH 1.2. However, as the orally ingested microparticles move from the stomach to the small intestine, release experiments by conducting the sequential pH change from the gastric pH to intestinal one will be needed for a more exact evaluation. 

### 2.5. In Vivo Efficacy of Ch-TBA-MP/Eud, Ch-TBA/TMC-MP/Eud, and Ch-MP/Eud after Intragastric Administration

**Figure 4:**
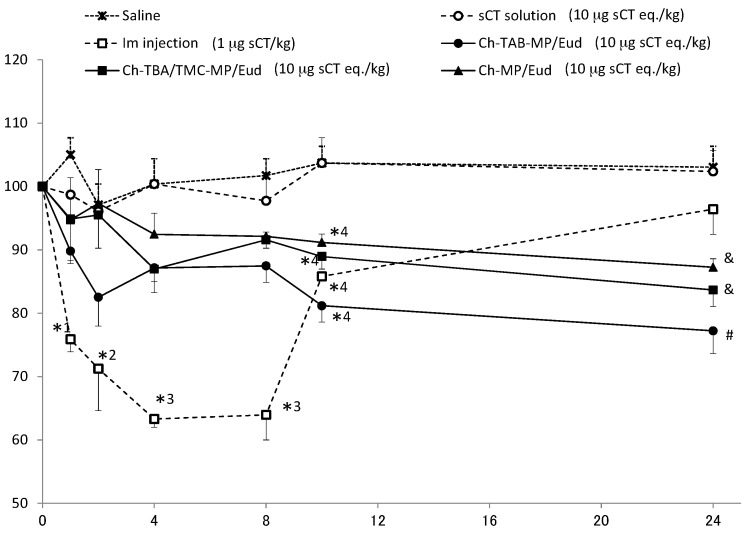
Plasma Ca levels after the intragastric administration of each preparation to rats. The initial level, defined as the level immediately before administration, was set to 100%. Each point represents the mean ± standard error of mean (S.E.M.) (*n* = 3). *^1^ *p* < 0.05 vs. saline, sCT solution, Ch-TBA/TMC-MP/Eud, Ch-MP/Eud; *^2^ *p* < 0.05 vs. saline, Ch-MP/Eud; *^3^ *p* < 0.05 vs. saline, sCT solution, Ch-TBA-MP/Eud, Ch-TBA/TMC-MP/Eud, Ch-MP/Eud; *^4^ *p* < 0.05 vs. saline, sCT solution; # *p* < 0.05 vs. saline, sCT solution, im injection; & *p* < 0.05 vs. saline, sCT solution (Tukey’s test).

Microparticles and solution containing sCT in saline were administered at a dose of 10 µg sCT eq./kg. Since the conventional administration of sCT is intramuscularly (i.m.), its saline solution was injected i.m. at a dose of 1 µg sCT eq./kg. Plasma Ca levels were monitored over time. Plasma concentrations immediately prior to administration were set to 100%. The results obtained are shown in Figure 4.

In the oral administration of sCT solution, plasma Ca levels were hardly reduced before the administration. They were very close to the levels by only saline (negative control). In the i.m. administration of sCT at 1 µg sCT eq./kg, plasma levels of Ca were rapidly eliminated, and reached the lowest level (63.3% of the initial level) 4 h after the i.m. injection. Plasma Ca levels recovered fairly rapidly from 8 h after the i.m. injection. All enteric-coated microparticles caused gradual decreases in plasma Ca levels. The rate and extent of the reductions observed in plasma Ca levels were the greatest in the order of Ch-TBA-MP/Eud > Ch-TBA/TMC-MP/Eud > Ch-MP/Eud. Ch-TBA-MP/Eud rapidly reduced Ca levels to 83%, and these levels continued and gradually decreased to 77% 24 h after the administration. 

Parameters depicting the degree of the reduction in plasma Ca levels were calculated using the mean values shown in Figure 4. The time required to reach the largest reduction in Ca levels, named T_R_, and the Ca level at T_R_, called C_R_, are shown in Table 3. The area between the initial Ca level (%) and above the curve of the plasma Ca level (%), named AAC, is equivalent to the area under the percent reduction-time curve [37,38]. AAC values were calculated for all preparations. The mean time for a reduction in Ca levels, called MRT, was also calculated. AAC and MRT were calculated by the trapezoidal method using the program MULTI [39]. AAC values were the largest in the order of i.m. injection > Ch-TBA-MP/Eud > Ch-TBA/TMC-MP/Eud > Ch-MP/Eud. However, the AAC values were not significantly different among Ch-TBA-MP/Eud, Ch-TBA/TMC-MP/Eud and Ch-MP/Eud. As to MRT, the i.m. injection had a short MRT value of 7.43 h. On the other hand, the MRT values of Ch-TBA-MP/Eud, Ch-TBA/TMC-MP/Eud, and Ch-MP/Eud were approximately two-fold that of the i.m. injection. Relative effectiveness, named RE, was calculated by the following equation [5,40]:

*RE* = *AAC* (preparation) × *Dose* (i.m.)/{*AAC* (i.m.) × *Dose* (preparation)} (1)

RE values are shown in Table 3. The results obtained indicated that Ch-TBA-MP/Eud had the greatest potential for reducing Ca levels among the microparticles tested. Although the ability of Ch-TBA-MP/Eud to reduce Ca levels was markedly less than that of i.m.-injected sCT, Ca levels were could be markedly reduced by a higher dose. Thus, when orally administered, Ch-TBA-MP/Eud were considered to improve the absorption of sCT to the greatest extent. Furthermore, Ch-TBA/TMC-MP/Eud and Ch-MP/Eud were better for oral absorption than the solution dosage form of sCT. These results suggest the potential of Ch-TBA-MP/Eud as a useful dosage form for the oral absorption of sCT.

## 3. Experimental Section

### 3.1. Materials

Chitosan (MW 200,000; Ch), 2,2′-dithiodipyridine (2-PDS), 1-methyl-2-pyrrolidinone, 2-iminothiolane, and Calcium C-test Wako kit were purchased from Wako Pure Chemical Industries, Ltd. (Osaka, Japan). Salmon calcitonin (sCT) was obtained from Funakoshi Co., Ltd. (Tokyo, Japan). Eudragit L100 was supplied from Rohm GmbH Chemische Fabrik (Darmstadt, Germany). Sorbitan sesquioleate (SO-15) was obtained from Nikko Chemicals Co., Ltd. (Tokyo, Japan). Somnopentyl was used for anesthesia as an injectable dosage form of sodium pentobarbital (Kyoritsu Seiyaku Corp., Tokyo, Japan). All other chemicals were of reagent grade.

### 3.2. Synthesis and Characterization of Trimethyl-Chitosan (TMC) and Chitosan-4-Thio-Butyl-Amidine (Ch-TBA)

#### 3.2.1. Synthesis and Characterization of TMC

TMC was produced by the reaction between Ch and iodomethane in a strong basic solution of NaOH as described by Sieval et al. [22,23]. Ch (1 g), NaI (2.4 g), and 5.5 mL of 15% (*w/v*) NaOH were added to a mixture of iodomethane (5.75 mL) and 1-metyl-2-pyrrolidinone (40 mL). The resultant mixture was refluxed on a water bath at 60 °C for 1 h. After the product was precipitated by the addition of ethanol, it was separated by centrifugation and washed with diethyl ether. The product was dissolved in 40 mL of 1-metyl-2-pyrrolidinone at 60 °C, and NaI (2.4 g), 5.5 mL of 15% (*w/v*) NaOH, and iodomethane (3.5 mL) were then added. The mixture was refluxed at 60 °C for 30 min. Iodomethane (1 mL) and NaOH (0.3 g) were added, and the mixture was stirred for 1 h. The reaction mixture was added to 20 mL of 10% (*w/v*) NaCl aqueous solution and stirred. The product was precipitated by the addition of 80% (*v/v*) ethanol aqueous solution, and separated by centrifugation. The addition of 10% (*w/v*) NaCl aqueous solution, followed by precipitation with ethanol, was repeated again. After the precipitate was dissolved in water, it was precipitated with ethanol. The precipitate was washed with diethyl ether and dried in vacuo to yield TMC.

The chemical structure of TMC obtained was examined by ^1^H-NMR spectroscopy, in which a JNM-LA500 (JEOL, Tokyo, Japan) was used as an NMR spectrometer and measurements were performed at 80 °C using D_2_O and 1% CD_3_COOD in D_2_O as solvents. The degree of *N*-trimethylation was calculated based on ^1^H-NMR spectra; the substitution degree was obtained from the ratio of the integrated intensity of *N*-trimethyl group protons (3.7–3.8 ppm) to that of the C1 proton (4.8–6 ppm) of the glucosamine unit of Ch [22,32].

#### 3.2.2. Synthesis and Characterization of Ch-TBA

Ch-TBA was synthesized using the method of Bernkop-Schnürch et al. [27]. Ch (300 mg) was dissolved in 30 mL of a 1% (*v/v*) acetic acid aqueous solution, and the pH of the solution was adjusted to 6 by adding 1 M NaOH aqueous solution. 2-Imminothiolane (120 mg) was added to the Ch solution, which was then stirred for 24 h. The resultant mixture was dialyzed using 5 mM HCl aqueous solution for 24 h, 5 mM HCl aqueous solution containing 1% (*w/v*) NaCl aqueous solution for 24 h, 5 mM HCl aqueous solution for 24 h, and 0.4 mM HCl aqueous solution for 24 h in this order. The remaining sample was lyophilized to yield Ch-TBA.

The Ch-TBA obtained was characterized for the content of the thiol group (–SH) using the 2-PDS method [33]. After Ch-TBA was dissolved in phosphate buffer of pH 4.5, 2-PDS was added, and the mixture was stirred at 30 °C for 30 min. The absorbance was measured at 324 nm. The content of the thiol group was calculated from the standard curve, which was obtained by measuring the cysteine standard solution. 

### 3.3. Preparation of sCT-Loaded Microparticles Using a Ch-TBA or Ch-TBA/TMC Mixture and Their Coating with Eudragit L100

#### 3.3.1. sCT-Loaded Ch-TBA-Based Microparticles (Ch-TBA-MP) or sCT-Loaded Ch-TBA/TMC-Based Microparticles (Ch-TBA/TMC-MP)

Both microparticles were prepared using a similar method, that is, W/O emulsification followed by the evaporation of the aqueous phase. The Ch-TBA or Ch-TBA/TMC (1:1, 1:4, and 4:1, *w/w*) mixture (80 mg) was dissolved in 1% (*v/v*) acetic acid aqueous solution. sCT (480 µg) was then added to the polymer solution. The resultant solution was dripped into 160 mL of liquid paraffin containing 1% (*w/v*) SO-15, which was being stirred at 500 rpm and sonicated at 45 kHz (100 W). After dripping finished, stirring with sonication was continued for 10 min. The resultant emulsion was stirred under reduced pressure at 30 °C and then at 40 °C. After the aqueous phase had evaporated sufficiently, evaporation was terminated. n-Hexane was added to the resultant mixture, and centrifugation was performed to precipitate the product. After washing with n-hexane, the product was dried in vacuo to yield Ch-TBA-MP and Ch-TBA/TMC-MP. As the control, sCT-loaded Ch microparticles (Ch-MP) were prepared in the same manner, except for the use of Ch as a polymer.

#### 3.3.2. Eudragit Coating of Ch-TBA-MP and Ch-TBA/TMC-MP

The enteric coating was conducted on Ch-TBA-MP, Ch-TBA/TMC-MP, and Ch-MP using Eudragit L100. The microparticles (50 mg) were dispersed and stirred in 1 mL of methanol solution containing Eudragit L100 (100 mg). The resultant suspension was added to 200 mL of 2% (*w/v*) SO-15-containing liquid paraffin, which was being stirred at 1000 rpm. The resultant mixture was stirred under reduced pressure to evaporate methanol. It was then stirred under reduced pressure at 40 °C. After n-hexane (200 mL) was mixed with the obtained suspension, the resultant mixture was centrifuged to separate the product. The precipitate was collected, washed in n-hexane, and dried in vacuo to yield Eudragit L100-coated Ch-TBA-MP, Ch-TBA/TMC-MP, and Ch-MP, named Ch-TBA-MP/Eud, Ch-TBA/TMC-MP/Eud, and Ch-MP/Eud, respectively.

### 3.4. Measurement of Particle Characteristics and sCT Contents

The particle shape and size of all types of microparticles were investigated using scanning electron microscopy (SEM) with a JEOL JSM-5600LV (JEOL, Tokyo, Japan) apparatus after the application of a thin platinum coating by a JEOL JFC-1600 auto fine coater. Particle shapes and surface structures were observed, and micrographs were taken. Particle size was examined by measuring the green diameters of 200 particles selected randomly from the micrographs, and mean sizes and size distribution were calculated. 

sCT contents were investigated in Eudragit L100-coated microparticles. The microparticles were broken and the concentration of sCT released was measured. In order to achieve this, the microparticles (2 mg) were placed in 1 mL of a 50% (*v/v*) methanol aqueous solution, and the mixture underwent sonication at 45 kz (100 W) for 30 min. After the treated mixture was centrifuged, the upper solution was filtered with a membrane filter (pore size of 0.45 µm). Protein concentrations in the filtrate were measured using the BCA protein assay kit (Funakoshi Co., Ltd., Tokyo, Japan) in order to determine sCT concentrations.

### 3.5. Examination of in Vitro Release from Eudragit-Coated Microparticles

The in vitro release profiles of Ch-TBA-MP/Eud, Ch-TBA/TMC-MP/Eud, and Ch-MP/Eud were investigated. JP 16 first fluid (pH 1.2) and second fluid (pH 6.8) were used as release media. The microparticles (6 mg) were added to 3 mL of incubation medium, and the mixture was incubated at 37 °C by horizontal shaking at 90 rpm. At 1, 4, 8, and 24 h, a 700 µL aliquot was collected and centrifuged, and 550 µL of the resultant supernatant was taken. The supernatant was further filtrated with a membrane filter (pore size of 0.45 µm), and the concentration of sCT in the filtrate was examined to obtain the released amount. The residue of the centrifuged sample tube was mixed with 550 µL of fresh medium, and the resultant suspension was returned to the incubation mixture. 

### 3.6. In Vivo Experiments on Gastrointestinal Absorption 

After rats were fasted for 24 h, microparticles were ingested by gastric intubation at a dose of 10 µg sCT eq./kg. Immediately before and 0.25, 0.5, 1, 2, 4, 8, and 24 h after their administration, blood samples (200 µL) were taken via the jugular vein under anesthesia with somnopentyl. After the centrifugation of blood samples, the Ca levels in the resultant plasma were measured using a Calcium C-test Wako kit. A plasma Ca level immediately before the administration was used as a baseline (100% of the Ca level). 

A saline solution of sCT was administered intragastrically at a dose of 10 µg sCT eq./kg for comparison. Furthermore, for comparisons with the oral administration route, an intramuscular injection of sCT saline solution was administered at a dose of 1 µg sCT eq./kg. In both animal experiments, blood sampling and the measurement of plasma Ca levels were performed in the same manner as that described for the microparticles. 

### 3.7. Statistical Analysis

Comparisons were performed using ANOVA followed by Tukey’s post-hoc test, and a significant difference was set as *p* < 0.05.

## 4. Conclusions

Microparticulate dosage forms loaded with sCT were prepared using Ch, Ch-TBA, and Ch-TBA/TMC mixtures as drug carriers. The microparticles obtained were coated with Eudragit L100 to give enteric-coated microparticles named Ch-MP/Eud, Ch-TBA-MP/Eud, and Ch-TBA/TMC-MP/Eud, respectively. These microparticles had sCT contents of 5.4, 4.1, and 2.7 µg/mg, respectively, and had similar particle features, namely, they were almost spherical in shape and had a mean size of 1.3–1.6 µm. All of the microparticles showed similar sCT release patterns, in which sCT was released gradually and release rates were slower at gastric pH (50%–60% with a 24 h incubation) than at intestinal pH (30%–50% after a 24 h incubation). In vivo studies with an intragastric administration route revealed that Ch-TBA-MP/Eud reduced plasma Ca levels the most among the microparticles tested. The relative effectiveness (RE) of Ch-TBA-MP/Eud was calculated to be 8.6% that of the i.m. injection of sCT. Ch-TBA/TMC-MP/Eud and Ch-MP/Eud showed RE of 6.6 and 5.1, respectively, while no reduction in plasma Ca levels was induced by the sCT solution. These results suggest that Ch-TBA-MP/Eud is superior to the other microparticles, and this may have been because Ch-TBA functions the most effectively to promote absorption due to its mucosal adsorption and loosening of the membrane barrier. The enteric-coated Ch-TBA microparticules are evaluated to have potential as a carrier system for the oral absorption of peptide drugs.

## Figures and Tables

**Table 1:** Formulations and particle formation and characteristics of Ch-TBA-MP, Ch-TBA/TMC-MP, and Ch-MP.

Preparation	Ch-TBA (mg)	TMC (mg)	Ch (mg)	sCT (µg)	Microparticle Formation	Size (nm)
Ch-TBA-MP	80	-	-	480	Formed	698 ± 258
Ch-TBA/TMC(4/1)-MP	64	16	-	480	Formed	778 ± 352
Ch-TBA/TMC(1/1)-MP	40	40	-	480	Not formed	-
Ch-TBA/TMC(1/4)-MP	16	64	-	480	Not formed	-
Ch-MP/Eud	-	-	80	480	Formed	654 ± 312

MP: microparticles; Ch-TBA: chitosan-4-thio-butylamidine conjugate; TMC: trimethyl-chitosan; Ch: chitosan; sCT: salmon calcitonin.

**Table 2:** Particle characteristics of Ch-TBA-MP/Eud, Ch-TBA/TMC-MP/Eud, **a**nd Ch-MP/Eud.

Preparation	sCT Concent (µg/mg)	Size (µm)
Ch-TBA-MP/Eud	4.1 ± 1.2	1.51 ± 1.23
Ch-TBA/TMC-MP/Eud *	2.7 ± 0.2	1.40 ± 1.60
Ch-MP/Eud	5.4 ± 0.8	1.35 ± 1.43

* Ch-TBA/TMC(4/1)-MP were used.

**Table 3:** Efficacy parameters of Ch-TBA-MP/Eud, Ch-TBA/TMC-MP/Eud and Ch-MP/Eud.

Preparation	Dose (µg sCT eq./kg)	C_R_ (% of the Initial Level)	T_R_ (h)	AAC (% × h)	MRT (h)	RE (%)
Saline	-	97.1	2	−58	14.7	−1.4
sCT solution	10	96.1	2	−34	18.9	−0.8
Im injection	1	63.3	4	424	7.4	100
Ch-TBA-MP/Eud	10	77.2	24	366	14.4	8.6
Ch-TBA/TMC-MP/Eud *	10	83.7	24	279	14.4	6.6
Ch-MP/Eud	10	87.3	24	215	14.6	5.1

Each parameter was obtained from the mean values shown in Figure 4. * Ch-TBA/TMC(4/1)-MP were used. T_R_: time required to reach the largest reduction in Ca level; C_R_: Ca level at T_R_; AAC: area between the initial Ca level (%) and above the curve of the plasma Ca level; MRT: mean time for a reduction in Ca level; RE: relative effectiveness, calculated by Equation (1). Im: intramuscular injection.
